# Adoption Readiness of AI-Based Robotic Surgery in Head and Neck Disciplines: A Cross-Sectional Multispecialty Analysis

**DOI:** 10.7759/cureus.110312

**Published:** 2026-06-05

**Authors:** Sunil Kumar Gulia, Sreenitha S Hosthor, Satish D Mehta, Deepak Kolte, Ifath Nazia Ghori, Hisham M Ibrahim

**Affiliations:** 1 Department of Oral and Maxillofacial Surgery, Shree Guru Gobind Singh Tricentenary University, Gurugram, IND; 2 Department of Forensic Odontology, Government Dental College and Research Institute, Bengaluru, IND; 3 Department of Orthopedics, Bharati Vidyapeeth (Deemed to be University) Medical College, Sangli, IND; 4 Department of Oral and Maxillofacial Surgery, Bharati Vidyapeeth (Deemed to be University) Dental College and Hospital, Mumbai, IND; 5 Department of Computer Science, Jazan University, Jazan, SAU; 6 Department of Oral and Maxillofacial Surgery, Al-Azhar Dental College, Thodupuzha, IND

**Keywords:** artificial intelligence, knowledge, perception, robotic surgery, surgeons

## Abstract

Introduction: AI-driven robotic surgery is increasingly being integrated into head and neck surgical practice. However, its clinical adoption depends on surgeons’ knowledge, practical exposure, and perception. This study aimed to assess the knowledge, practice, and perception of AI-driven robotic surgery and evaluate the knowledge-practice gap among surgeons.

Materials and methods: A cross-sectional, questionnaire-based analytical study was conducted among 150 surgeons involved in head and neck procedures, including oral and maxillofacial surgeons (n = 42), otorhinolaryngologists (n = 38), oncologic surgeons (n = 35), and general surgeons (n = 35). A validated questionnaire assessed knowledge (six items), practice (five items), and perception (nine items). Data were analyzed statistically, with descriptive and inferential statistics. Confirmatory factor analysis and correlation analyses were performed.

Results: High levels of knowledge were observed across specialties, with correct identification of AI-driven robotic surgery reported by 35 (83.3%) oral and maxillofacial surgeons, 30 (78.9%) otorhinolaryngologists, 32 (91.4%) oncologic surgeons, and 27 (77.1%) general surgeons. Despite this, practical exposure was limited; formal training was reported by 18 (42.9%), 14 (36.8%), 20 (57.1%), and 12 (34.3%) participants. Frequent involvement in robotic procedures was low across groups, with only eight (19.0%), six (15.8%), 12 (34.3%), and five (14.3%) surgeons reporting regular use. The perception of robotic surgery was positive, with high agreement regarding improved surgical precision and patient safety. However, high cost and lack of training were identified as major barriers. A significant knowledge-practice gap was observed across all specialties (p < 0.001). Correlation analysis demonstrated significant positive associations between knowledge and practice (r = 0.387), knowledge and perception (r = 0.341), and practice and perception (r = 0.462) (p < 0.001).

Conclusion: Surgeons demonstrate high knowledge and a favorable perception of AI-driven robotic surgery; however, limited practical exposure highlights a significant knowledge-practice gap. Enhancing structured training programs and improving accessibility to robotic systems are essential for effective clinical integration.

## Introduction

AI-driven robotic surgery represents a transformative advancement in modern surgical practice, particularly in the field of head and neck procedures [1]. By integrating machine learning, real-time image guidance, and enhanced precision, robotic systems have the potential to improve surgical outcomes, reduce intraoperative errors, and enhance patient safety [2]. These systems are increasingly being explored across multiple specialties, including oral and maxillofacial surgery, otorhinolaryngology, oncology, and general surgery [3]. Despite rapid technological progress, the clinical adoption of AI-assisted robotic surgery remains variable, often influenced by factors such as training availability, institutional resources, and surgeon acceptance [4].

Understanding the readiness of surgeons to adopt such technologies is crucial for successful integration into routine clinical practice. Previous evidence suggests that while awareness and theoretical knowledge of robotic surgery may be high, actual utilization and hands-on experience are often limited [5,6]. This discrepancy highlights the presence of a knowledge-practice gap, which may act as a significant barrier to widespread implementation. Furthermore, perceptions regarding cost, training requirements, and the potential impact on surgical autonomy can influence acceptance and future adoption [4].

The present study was designed as a cross-sectional, questionnaire-based analytical investigation involving surgeons from multiple specialties engaged in head and neck procedures. The aim of this study was to assess the knowledge, practice, and perception of AI-driven robotic surgery among head and neck surgeons across multiple specialties. The objectives were to evaluate the level of awareness and understanding, assess the extent of clinical exposure and utilization, analyze perception and acceptance toward robotic surgery, and identify the knowledge-practice gap and its associated factors.

## Materials and methods

Study design and setting

This cross-sectional, questionnaire-based analytical study was conducted in the Department of Oral and Maxillofacial Surgery, Bharati Vidyapeeth (Deemed to be University) Dental College and Hospital, Navi Mumbai, India. The study was carried out over a period of four months from September 2023 to December 2023, using an electronically administered survey. It specifically included oral and maxillofacial surgeons involved in the head and neck surgical procedures. This study was conducted after obtaining ethical approval (BVDUDC&H-IEC-560/2023) from the institutional ethics committee.

Study population and sampling

The study population comprised surgeons with at least one year of postgraduate clinical experience and active involvement in head and neck surgical procedures, encompassing residents, fellows, and consultants across multiple specialties. Only those who provided written informed consent were included. A non-probability convenience sampling strategy was employed, given the cross-sectional, survey-based nature of the study and the exploratory objectives of the investigation.

Sample size estimation was performed a priori using G*Power software (version 3.1.9.7; Heinrich-Heine-Universität Düsseldorf, Düsseldorf, Germany). Based on an anticipated medium effect size (Cohen's f = 0.25), derived from comparable survey-based studies assessing surgical knowledge and technology adoption, a two-tailed significance level of α = 0.05, and a desired statistical power of 1 − β = 0.80 (80%), the minimum required sample size was calculated to be 128 participants. To account for an anticipated non-response and incomplete questionnaire rate of approximately 15%, based on previously reported attrition in similar cross-sectional surgical surveys, the target sample was adjusted upward to 150 participants.

Questionnaire development and validation

A structured questionnaire was developed after an extensive review of the literature on AI-driven robotic surgery. The instrument includes three domains: knowledge (six items), practice (five items), and perception (nine items) (Appendix A). Content validity was assessed by a panel of experts in the field of oral and maxillofacial surgery, research methodology, and biostatistics, and necessary modifications were made to improve the clarity and relevance.

A pilot study was conducted with 20 participants to evaluate feasibility and comprehensibility. The internal consistency of the perception domain was assessed using Cronbach’s alpha, which showed strong reliability (α = 0.847). Construct validity was further established through confirmatory factor analysis, which demonstrated acceptable factor loadings and good model fit indices.

Data collection procedure

Data were collected using a self-administered electronic questionnaire distributed via email and messaging platforms. Participation was voluntary, and confidentiality was maintained. No personal identifiers were collected, and all responses were anonymized prior to analysis.

Outcome measures and assessment of the questionnaire

The primary outcome measures were the knowledge, practice, and perception scores. The knowledge score was calculated as the sum of correct responses in the knowledge domain, with higher scores indicating greater awareness. The practice score was assessed based on the participants’ exposure to robotic surgery, including training, frequency of use, institutional availability, confidence level, and utilization of AI-based tools in surgical planning. The perception score was derived from Likert-scale responses, with higher scores reflecting more favorable attitudes toward AI-driven robotic surgery. To evaluate the discrepancy between theoretical knowledge and real-world clinical applications, a knowledge-practice gap was calculated. This gap was defined as the difference between the standardized knowledge and practice scores, and a gap index was computed to quantify underutilization despite awareness.

Statistical analysis

Data were entered and analyzed using IBM SPSS Statistics for Windows, version 26.0 (IBM Corp., Armonk, NY, USA), and supplementary analyses were performed using R software (R Foundation for Statistical Computing, Vienna, Austria) for advanced modeling. Descriptive statistics are expressed as mean ± standard deviation for continuous variables and as frequencies (n) and percentages (%) for categorical variables. The normality of the data distribution was assessed using the Shapiro-Wilk test. For intergroup comparisons, one-way analysis of variance (ANOVA) was applied for normally distributed continuous variables, whereas the Kruskal-Wallis test was used for non-parametric data. Categorical variables were compared using the chi-square test.

The knowledge-practice gap was evaluated using paired t-tests to compare standardized knowledge and practice scores within participants. Correlations among knowledge, practice, and perception scores were assessed using Pearson’s correlation coefficient. A confirmatory factor analysis (CFA) was performed to validate the perception domain. Model fit was evaluated using indices including the comparative fit index, Tucker-Lewis index, root mean square error of approximation, and standardized root mean square residual. Factor loadings ≥0.5 were considered acceptable. Statistical significance was set at P <0.05.

## Results

A total of 150 surgeons participated in the study, including 42 (28%) oral and maxillofacial surgeons, 38 (25.4%) otorhinolaryngologists, 35 (23.3%) oncologic surgeons, and 35 (23.3%) general surgeons. The demographic distribution was comparable across the groups, with the majority being male and having six to 10 years of clinical experience. Most participants were affiliated with teaching hospitals, and there were no statistically significant intergroup differences (Table 1).

**Table 1:** Sociodemographic characteristics of participants (n = 150). Values are expressed as mean ± standard deviation or frequency (n, %). OMFS: oral and maxillofacial surgeons; ENT: otorhinolaryngologists

Characteristic	Category	OMFS (n = 42)	ENT (n = 38)	Oncologic surgeons (n = 35)	General surgeons (n = 35)
Age (years), mean ± SD		34.2 ± 5.8	36.1 ± 6.3	38.4 ± 7.1	35.7 ± 5.4
Sex, n (%)	Male	28 (66.7)	24 (63.2)	23 (65.7)	22 (62.9)
	Female	14 (33.3)	14 (36.8)	12 (34.3)	13 (37.1)
Experience (years), n (%)	1–5 years	15 (35.7)	12 (31.6)	10 (28.6)	13 (37.1)
	6–10 years	16 (38.1)	15 (39.5)	14 (40.0)	12 (34.3)
	>10 years	11 (26.2)	11 (28.9)	11 (31.4)	10 (28.6)
Institution type, n (%)	Teaching hospital	31 (73.8)	27 (71.1)	25 (71.4)	24 (68.6)
	Private hospital	11 (26.2)	11 (28.9)	10 (28.6)	11 (31.4)

Participants demonstrated a high level of knowledge regarding AI-driven robotic surgery, with the most accurate identification of its principles and applications. Awareness regarding the necessity of training and the role of AI in enhancing surgical precision was consistently high across all groups without significant differences (Table 2).

**Table 2:** Knowledge-related responses regarding AI-driven robotic surgery across surgical specialties. Values are expressed as frequency (n, %). The chi-square test (χ²) was used and p < 0.05 was considered statistically significant. OMFS: oral and maxillofacial surgeons, ENT: otorhinolaryngologists

Knowledge item	OMFS n (%)	ENT n (%)	Oncologic surgeons n (%)	General surgeons n (%)	Test stats	p-value
Defines AI-driven robotic surgery correctly	35 (83.3)	30 (78.9)	32 (91.4)	27 (77.1)	χ²=3.84	0.279
AI primarily helps in decision/image/precision	36 (85.7)	31 (81.6)	33 (94.3)	28 (80.0)	χ²=4.21	0.240
Robotic systems widely used in head and neck surgery	28 (66.7)	25 (65.8)	28 (80.0)	22 (62.9)	χ²=3.16	0.367
AI can reduce intraoperative errors	37 (88.1)	34 (89.5)	33 (94.3)	29 (82.9)	χ²=2.14	0.544
Training mandatory before robotic surgery	40 (95.2)	36 (94.7)	35 (100%)	33 (94.3)	χ²=1.98	0.577
AI-based systems learn from surgical data	32 (76.2)	27 (71.1)	30 (85.7)	24 (68.6)	χ²=4.08	0.253

However, the practical exposure to robotic surgery is limited. Less than half of the participants reported formal training, and only a minority were frequently involved in robotic procedures or routinely used AI-based tools in surgical planning. Although a relatively higher exposure was observed among oncologic surgeons, these differences were not statistically significant (Table 3).

**Table 3:** Practice-related responses regarding AI-driven robotic surgery across surgical specialties. Values are expressed as frequency (n, %). The chi-square(χ²)  test was applied and p < 0.05 was considered statistically significant. OMFS: oral and maxillofacial surgeons, ENT: otorhinolaryngologists

Practice item	OMFS n (%)	ENT n (%)	Oncologic surgeons n (%)	General surgeons n (%)	Test stats	p-value
Received formal robotic surgery training (Yes)	18 (42.9)	14 (36.8)	20 (57.1)	12 (34.3)	χ²=5.63	0.131
Assisted/performed robotic surgery (Frequently)	8 (19.0)	6 (15.8)	12 (34.3)	5 (14.3)	χ²=7.21	0.065
Institution has robotic system access (Yes)	22 (52.4)	19 (50.0)	24 (68.6)	17 (48.6)	χ²=4.89	0.180
Confidence in robotic system use (≥4/5)	14 (33.3)	11 (28.9)	17 (48.6)	9 (25.7)	χ²=6.14	0.105
AI tools in surgical planning (Often/Always)	10 (23.8)	9 (23.7)	15 (42.9)	8 (22.9)	χ²=5.77	0.123

Perceptions of AI-driven robotic surgery were generally positive across all specialties. The participants strongly agreed on its benefits in improving surgical precision and patient safety. However, high costs and a lack of training have been identified as major barriers. Significant differences were observed in attitudes toward the integration of robotic surgery into training programs and willingness to adopt technology (p<0.05) (Table 4).

**Table 4:** Perception-related responses regarding AI-driven robotic surgery across surgical specialties. Values represent mean ± standard deviation (SD) on a 5-point Likert scale (1=Strongly disagree, 5=Strongly agree). One-way analysis of variance (ANOVA) (F) was used for normally distributed items, Kruskal–Wallis H was substituted where Levene's test indicated heterogeneity of variance. *p <0.05 was considered significant. OMFS: oral and maxillofacial surgeons, ENT: otorhinolaryngologists

Perception item	OMFS	ENT	Oncologic surgeons	General surgeons	Test stats	p-value
AI-driven robotic surgery improves surgical precision	4.62 ± 0.51	4.58 ± 0.54	4.74 ± 0.45	4.49 ± 0.58	F=2.11	0.101
It reduces surgeon fatigue	4.31 ± 0.72	4.22 ± 0.78	4.43 ± 0.67	4.18 ± 0.81	F=1.47	0.224
It enhances patient safety	4.55 ± 0.58	4.49 ± 0.62	4.66 ± 0.50	4.41 ± 0.69	F=2.04	0.110
High-cost limits widespread adoption	4.71 ± 0.46	4.68 ± 0.49	4.80 ± 0.41	4.63 ± 0.55	F=1.88	0.135
Lack of training is a major barrier	4.64 ± 0.53	4.61 ± 0.57	4.77 ± 0.43	4.57 ± 0.60	F=2.37	0.072
AI may reduce surgeon decision-making autonomy	3.48 ± 0.94	3.52 ± 0.97	3.37 ± 1.01	3.61 ± 0.89	F=0.89	0.447
Robotic surgery should be integrated into training programs	4.69 ± 0.48	4.63 ± 0.52	4.83 ± 0.38	4.57 ± 0.61	H=8.74	0.033*
I am willing to adopt robotic surgery in my practice	4.21 ± 0.81	4.14 ± 0.87	4.40 ± 0.70	4.06 ± 0.92	F=2.78	0.043*
AI-driven robotic surgery = future of head and neck procedures	4.57 ± 0.55	4.51 ± 0.60	4.69 ± 0.47	4.44 ± 0.66	F=2.26	0.084

**Figure 1:**
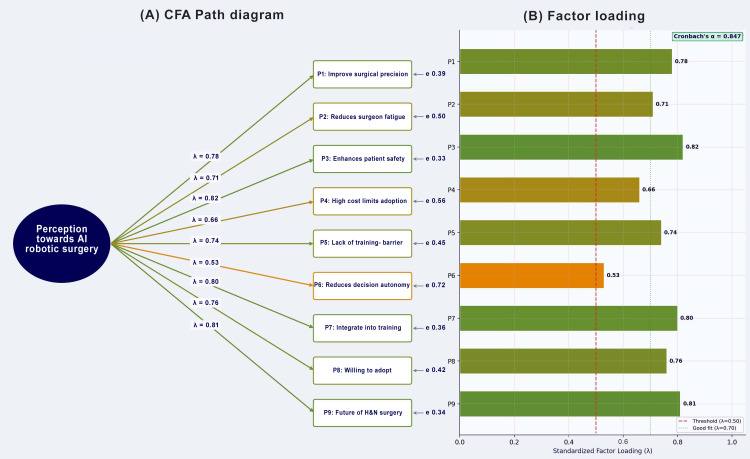
CFA (A) path diagram; (B) factor loading. Factor loadings λ are presented for each item. In Panel (A), loadings range from 0.53 to 0.81; Panel (B) reports Cronbach's α for each item, with values in bold indicating acceptable internal consistency where α ≥ 0.70. The dashed line at λ = 0.50 denotes the minimum threshold for item retention in factor analysis, while the solid line at λ ≥ 0.70 indicates strong factor loading. CFA: confirmatory factor analysis

Confirmatory factor analysis supported the construct validity of the perception domain, demonstrating acceptable factor loadings and good model fit (Figure 1).

**Figure 2:**
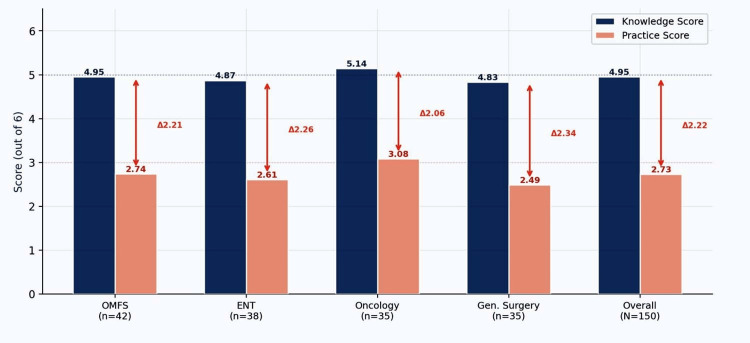
Knowledge and practice score by speciality. Data values are presented as the mean score of knowledge and practice, and gap analysis is presented as Δ mean value. OMFS: oral and maxillofacial surgeons, ENT: otorhinolaryngologists; Gen: general

`A significant knowledge-practice gap was observed across all groups, with knowledge levels exceeding practical implementation (p < 0.001), indicating the underutilization of robotic systems despite adequate awareness (Figure 2).

Pearson correlation analysis revealed significant positive relationships between knowledge, practice, and perception scores, suggesting that greater exposure to robotic systems is associated with more favorable attitudes toward their adoption (Table 5). Overall, while the knowledge and perception regarding AI-driven robotic surgery were high, its clinical implementation remained limited, highlighting a substantial gap between awareness and practice.

**Table 5:** Correlation between knowledge, practice, and perception scores. Values are Pearson correlation coefficients (r) with corresponding two-tailed p‑values. *Indicates statistical significance at p < 0.001. Pearson correlation coefficients (r) range from -1 to +1. Values closer to +1 indicate a stronger positive linear relationship.

Variable pair	r	p-value	Interpretation
Practice score vs. Knowledge score	0.387	< 0.001*	Weak positive
Perception score vs. Knowledge score	0.341	< 0.001*	Weak positive
Perception vs. Practice score	0.462	< 0.001*	Moderate positive

## Discussion

The present study evaluated the knowledge, practice, and perception of AI-driven robotic surgery among surgeons involved in head and neck procedures and revealed three key findings: high levels of knowledge, favorable perception, and limited practical exposure, resulting in a significant knowledge-practice gap. These findings highlight the transitional stage in the adoption of advanced surgical technologies.

The knowledge domain demonstrated that the majority of participants had a good understanding of AI-driven robotic surgery, particularly regarding its role in improving surgical precision, decision-making, and reducing intraoperative errors. These findings are consistent with those of previous studies reporting an increasing awareness of artificial intelligence among clinicians [7,8]. However, knowledge appeared to be stronger for basic concepts than for broader clinical applications, suggesting that the current understanding remains largely theoretical [9]. This reflects the need for more structured application-oriented training.

The perception domain showed a generally positive attitude toward AI-driven robotic surgery across all specialties. The participants strongly agreed that robotic surgery improves surgical precision and patient safety. The positive perception observed in the present study is consistent with the findings of Al Dihan et al. [10], who reported favorable attitudes and high expectations for robotic surgery among patients attending surgical specialty clinics, highlighting the growing acceptance of advanced surgical technologies across both clinicians and patients. Similar results were reported by Stai et al. [11] and Aldousari et al. [12]. However, high costs and lack of training were consistently identified as major barriers, supporting earlier reports that economic and educational limitations significantly hinder implementation, particularly in developing healthcare systems [13]. Boys et al. [14] demonstrated that although most individuals perceive robotic surgery as beneficial, significant misconceptions persist regarding its functionality, including beliefs about the partial autonomy of robotic systems. This aligns with the moderate concerns regarding surgeon autonomy observed in the present study, highlighting the need for improved education and clarity regarding the role of surgeons in AI-assisted procedures.

Despite high knowledge levels, the practice domain has limited clinical implementation. Less than half of the participants reported formal training, and only a small proportion were frequently involved in robotic procedures or routinely used AI-based tools. The limited formal training observed in the present study is consistent with the findings of Farivar et al. [15], who reported that although many surgical residents are exposed to robotic systems, a substantial proportion receive no structured training prior to participation in robotic procedures. This highlights a critical gap in surgical education and supports the need for standardized training protocols to facilitate the effective integration of robotic surgery into clinical practice.

Although oncologic surgeons demonstrated relatively higher exposure, the differences across specialties were not statistically significant, indicating that these limitations are widespread. The high level of awareness observed in the present study is consistent with findings by Chan et al. [16], who reported that clinicians generally demonstrate adequate awareness and positive attitudes toward robotic surgery, although variability exists in the depth of understanding across domains. However, similar to the present findings, gaps in detailed knowledge and application-oriented understanding have been reported.

A key finding of this study was the presence of a significant knowledge-practice gap. While knowledge levels were consistently high, practical applications remained limited, indicating underutilization of available knowledge. Similar gaps have been reported in prior studies, emphasizing that awareness alone is insufficient for adoption without adequate infrastructure and hands-on training [5,6].

Furthermore, correlation analysis demonstrated significant positive relationships among knowledge, practice, and perception scores. The moderate correlation between practice and perception suggests that increased exposure to robotic systems is associated with more favorable attitudes toward their use. This finding reinforces the importance of experiential learning and aligns with previous studies that highlight the role of training in improving acceptance of emerging technologies [17,18]. Confirmatory factor analysis supported the validity of the perception domain, indicating that the questionnaire reliably captured attitudes toward AI-driven robotic surgery. This strengthens the methodological robustness of the study.

Clinical implications and limitations

The findings of this study have several important clinical implications. The combination of high knowledge and positive perception suggests that surgeons are receptive to AI-driven robotic surgery; however, practical barriers limit its adoption. The integration of structured training programs, simulation-based learning, and hands-on workshops into surgical education is essential for bridging the knowledge-practice gap. Improving accessibility to robotic systems and addressing cost-related challenges are critical for wider implementation. Additionally, incorporating AI-focused modules into postgraduate curricula may facilitate a smooth transition from awareness to clinical application.

However, the limitations of this study must be acknowledged. The cross-sectional design limits causal inferences, and the use of convenience sampling may affect generalizability. Self-reported responses may have introduced reporting bias, and actual clinical competency was not assessed. Although multiple specialties were included, these findings may not fully represent all surgical disciplines. Future multicenter and longitudinal studies are recommended to validate these findings and to evaluate the impact of targeted training interventions.

## Conclusions

The present study demonstrated that surgeons involved in head and neck procedures possessed high levels of knowledge and generally favorable perceptions of AI-driven robotic surgery. However, practical exposure and formal training remain limited, resulting in a significant knowledge-practice gap. While clinicians have recognized the potential benefits of improved precision and patient outcomes, barriers such as high cost and lack of structured training have hindered widespread adoption. The positive correlation among knowledge, practice, and perception highlights the importance of experiential learning. Strengthening training programs, improving accessibility, and integrating AI education into surgical curricula are essential for facilitating effective clinical implementation.

## Appendices

Appendix A

Study Questionnaire

**Table 6:** Questionnaire for demographic details of study population.

Demographic		
Name		
Age		
Gender	Male	
	Female	
Speciality	Oral and maxillofacial surgeon	
	Otorhinolaryngologist (ENT)	
	Head and neck oncology (Oncologist)	
	General Surgeon	
Experience (years)	1-5	
	5-10	
	>10	

**Table 7:** Questionnaire for knowledge, practice and attitude towards AI-based robotic surgery in head and neck disciplines.

Part 1- KNOWLEDGE (6 ITEMS)							Response
1	AI-driven robotic surgery refers to						
	a) Fully autonomous surgery b) Surgeon-assisted robotic systems enhanced with AI c) Manual surgery with imaging d) Not sure						
2	AI in robotic surgery primarily helps in						
	a) Decision support b) Image guidance c) Precision enhancement d) All of the above						
3	Robotic systems are currently widely used in head and neck surgery						
	True False Not sure						
4	AI can reduce intraoperative errors						
	True False Not sure						
5	Training is mandatory before performing robotic surgery						
	True False Not sure						
6	AI-based systems can learn from surgical data over time						
	True False Not sure						
Part 2- PRACTICE (5 ITEMS)							Response
1	Have you received formal training in robotic surgery						
	Yes No						
2	Have you assisted or performed robotic surgery						
	Never Occasionally Frequently						
3	Does your institution have access to robotic surgical systems						
	Yes No Not sure						
4	How confident are you in using robotic systems						
	Very low Low Average High Very high						
5	How often do you incorporate AI-based tools in surgical planning						
	Never Occasionally Frequently Always						
Part 2- PERCEPTION (9 ITEMS)							
1		AI-driven robotic surgery improves surgical precision					
		Strongly disagree	Disagree	Neutral	Agree	Strongly agree	
2		It reduces surgeon fatigue					
		Strongly disagree	Disagree	Neutral	Agree	Strongly agree	
3		It enhances patient safety					
		Strongly disagree	Disagree	Neutral	Agree	Strongly agree	
4		High cost limits its widespread adoption					
		Strongly disagree	Disagree	Neutral	Agree	Strongly agree	
5		Lack of training is a major barrier					
		Strongly disagree	Disagree	Neutral	Agree	Strongly agree	
6		AI may reduce the surgeon’s decision-making autonomy					
		Strongly disagree	Disagree	Neutral	Agree	Strongly agree	
7		Robotic surgery should be integrated into surgical training programs					
		Strongly disagree	Disagree	Neutral	Agree	Strongly agree	
8		I am willing to adopt robotic surgery in my practice					
		Strongly disagree	Disagree	Neutral	Agree	Strongly agree	
9		AI-driven robotic surgery represents the future of head and neck procedures					
		Strongly disagree	Disagree	Neutral	Agree	Strongly agree	
